# Supplementary material for: BK channel inactivation gates daytime excitability in the circadian clock
Source: Nat Commun. 2016 Mar 4;7:10837. doi: 10.1038/ncomms10837 (PMC4785228; doi:10.1038/ncomms10837)
Supplement: Supplementary Information — Supplementary Figures 1-9, Supplementary Table 1, Supplementary Discussion and Supplementary References. [file ncomms10837-s1.pdf]

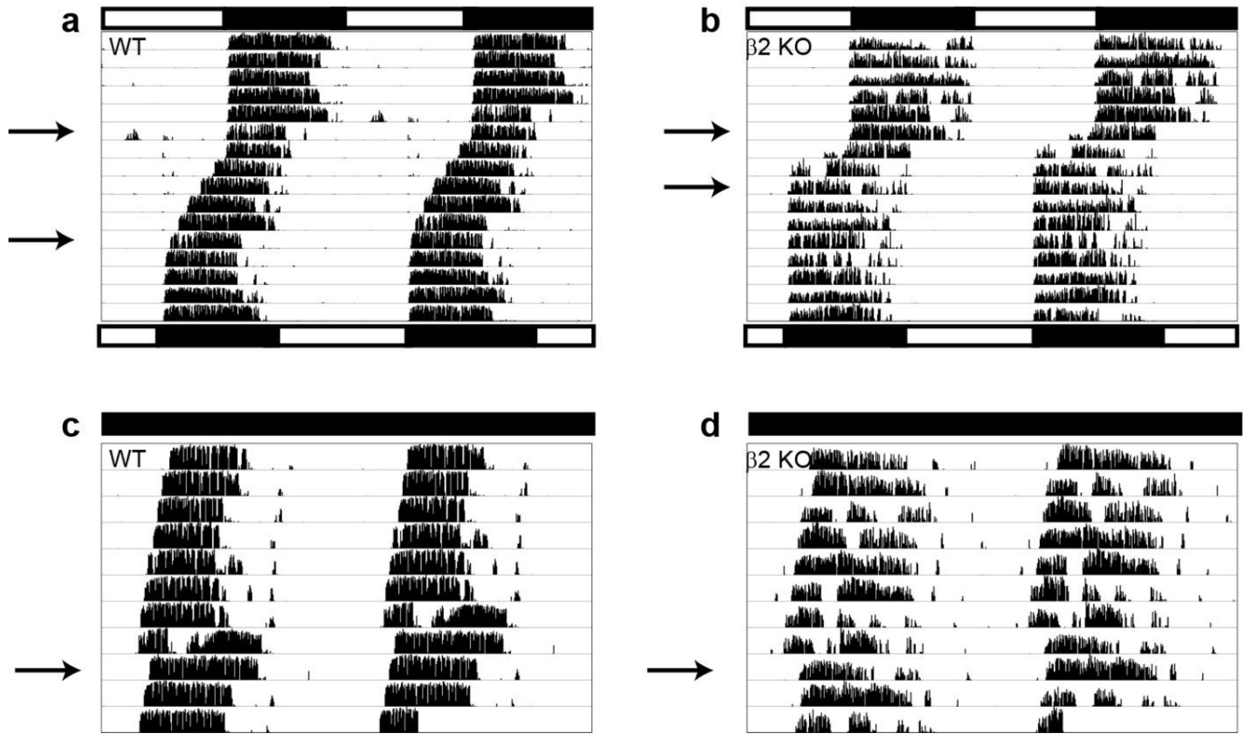

Supplementary Figure 1. The β2 subunit is required for normal re-entrainment and phase-shifting behavior. (**a,b**) Representative actograms from re-entrainment protocol (see Methods). (**a**) Locomotor wheel running activity from a WT mouse before (light:dark cycle at top) and after a 6 hr phase advance (light:dark cycle at bottom). First arrow denotes day of light:dark cycle advance, and the second arrow denotes the first day of stable re-entrainment (6 days to re-entrain). (**b**) β2 KO actogram (3 days to re-entrain). (**c,d**) Representative actograms from phase-shift protocol. (**c**) WT mouse in DD. A 30 min light pulse was delivered at CT16 on the day indicated by the arrow (1.6 hr phase delay). (**d**) β2 KO actograms (2.5 hr phase delay). Group average values provided in Fig. 1i,j and Supplementary Table 1.

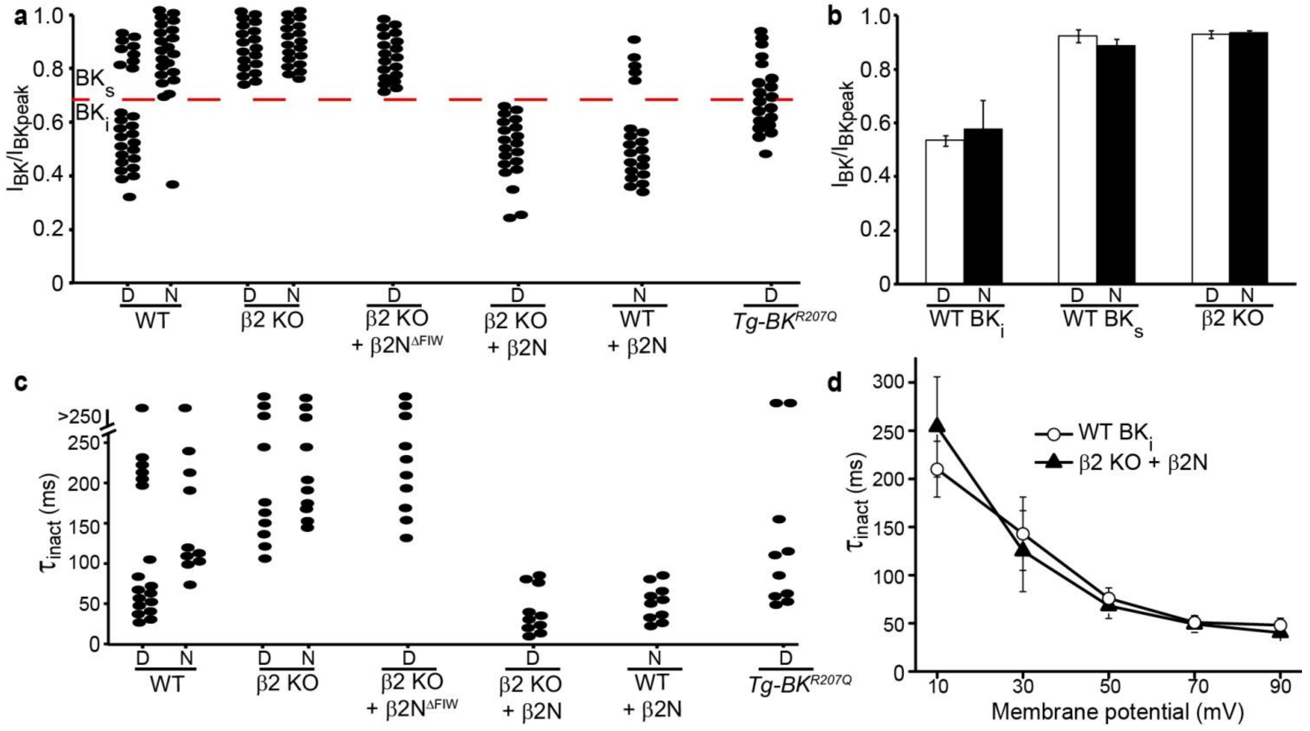

Supplementary Figure 2. Cross-comparison of fractional current and τ_{inact} values across all conditions. (**a**) Fractional BK current values (I_{BK}/I_{BKpeak}). Line denotes 0.7. BK_i < 0.7 and BK_s > 0.7. Number of neurons for each condition: WT, day (27) and night (22); β2 KO, day (20) and night (20); β2 KO + β2N$^{\Delta FIW}$, day (18); β2 KO + β2N, day (20); WT + β2N, night (20); and *Tg-BK*^{R207Q}, day (20). (**b**) Average fractional current values at +90 mV for BK_i and BK_s currents from WT SCNs. All β2 KO currents were BK_s. *n's*: WT, day BK_i (18), BK_s (9) and night BK_i (3) BK_s (19); β2 KO, day (20) and night (20). (**c**) τ_{inact} values at +90 mV. *n's*: WT, day (18) and night (12); β2 KO, day (10) and night (10); β2 KO + β2N$^{\Delta FIW}$, day (10); β2 KO + β2N, day (10); WT + β2N, night (10); and *Tg-BK*^{R207Q}, day (13). Values are mean ± SEM. (**d**) There was no difference in the τ_{inact} values for BK_i currents versus β2 KO + β2N.

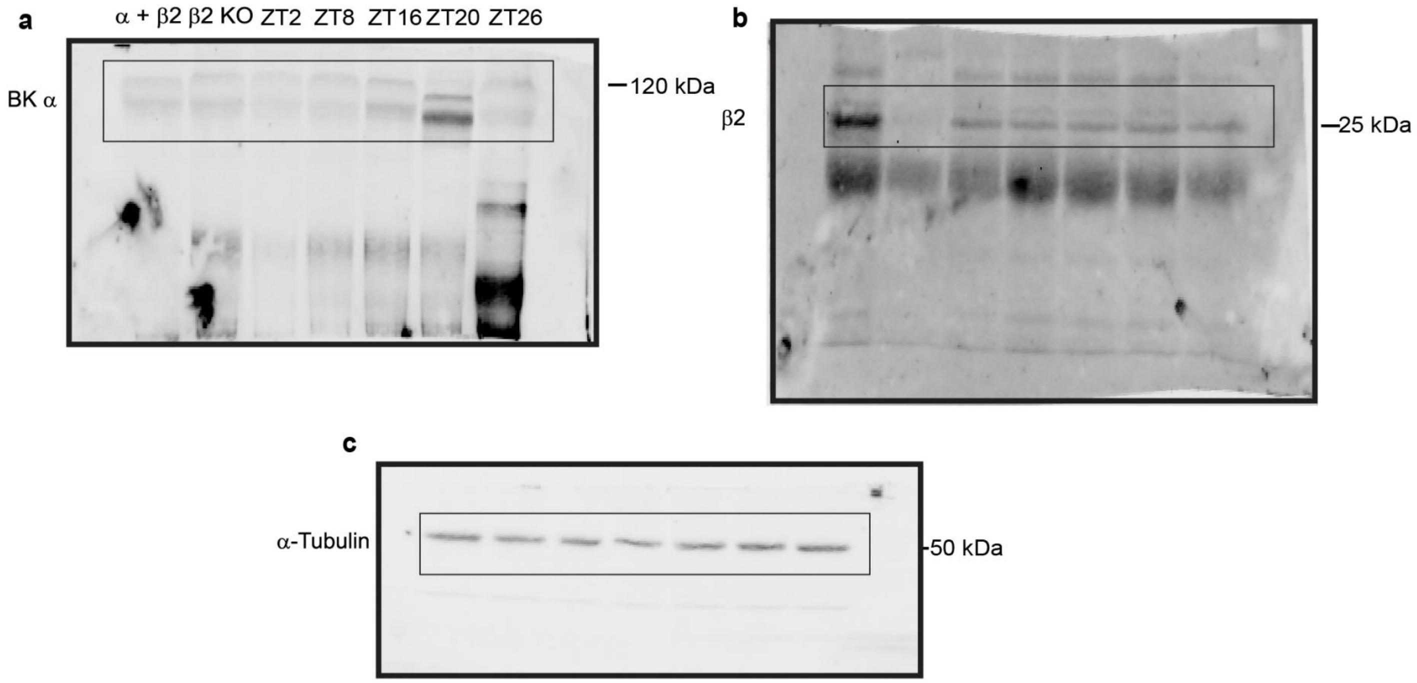

Supplementary Figure 3. Western blot membranes for Main Figure 3. Full membrane images for representative panels displayed in Main Figure 3 (inner boxes). Thick outer outlines indicate the border of each membrane.

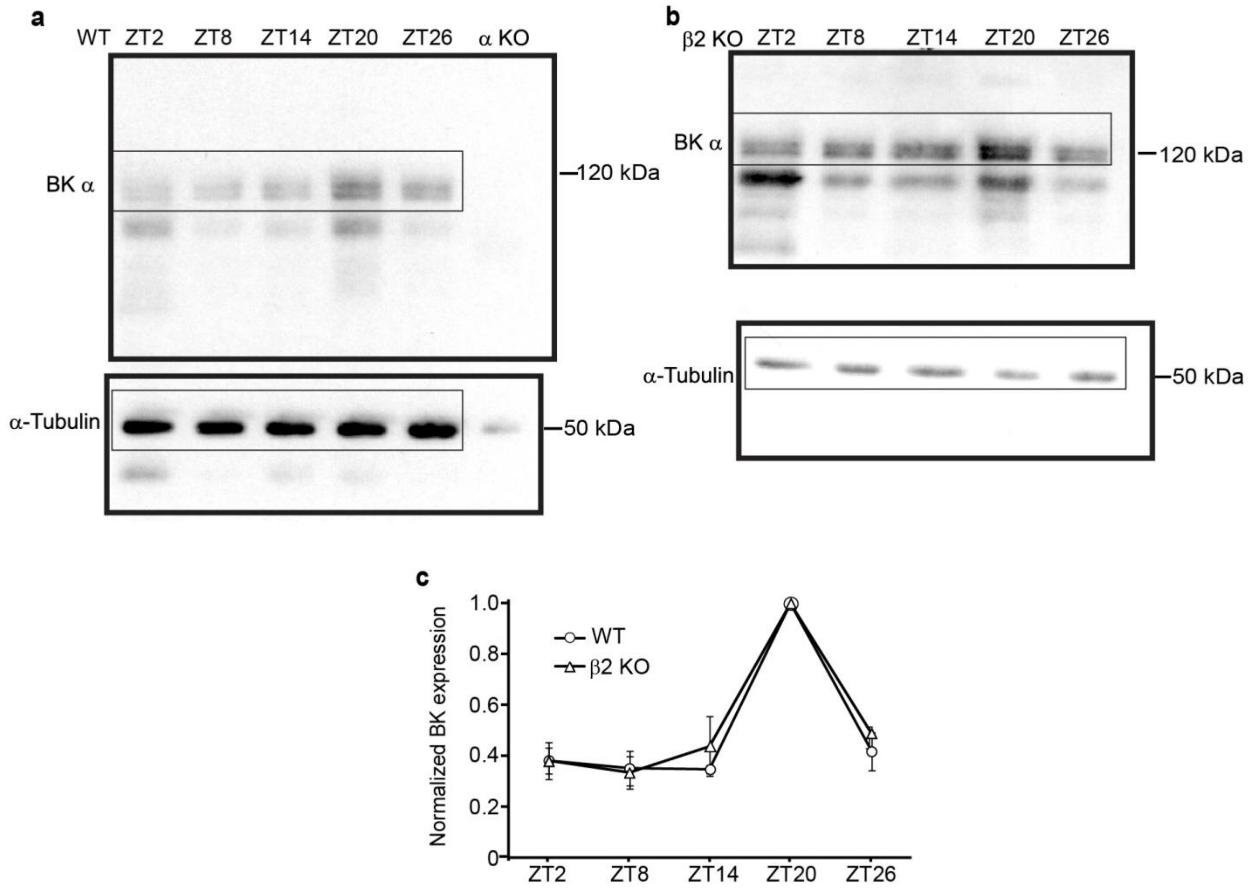

Supplementary Figure 4. Expression of BK α in WT and β2 KO SCNs. (**a**) Representative western blots (full membrane shown) showing BK α subunit expression in WT SCNs harvested at 6 hr intervals (top). α-tubulin expression was used as a loading control (bottom). (**b**) BK α subunit (top) and α-tubulin (bottom) expression from β2 KO SCNs. (**c**) BK α band intensity normalized to α-tubulin and plotted as a proportion of ZT20 expression. BK α expression was not different in β2 KO compared to WT (*P* = 0.47, Two-Way ANOVA). Data are the average from 3 independent timed SCN collections (3 SCNs per genotype at each timepoint).

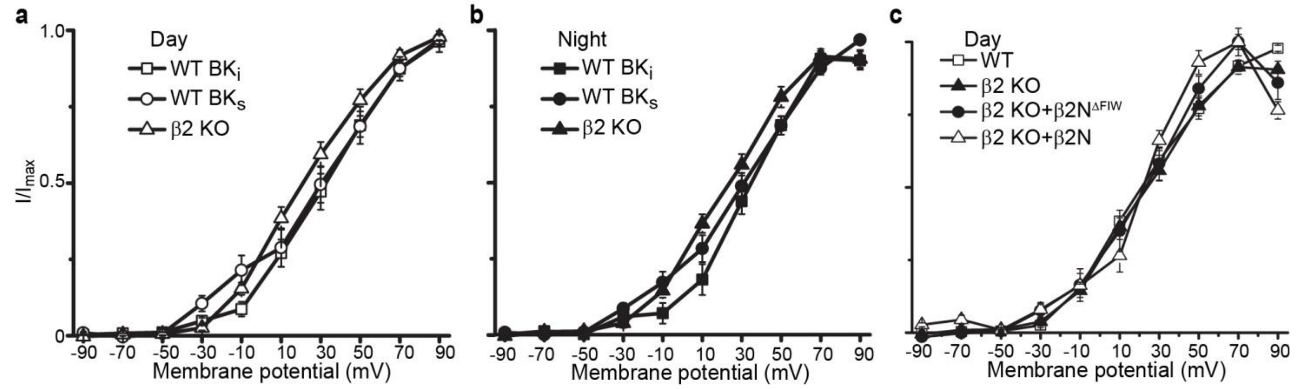

Supplementary Figure 5. Normalized current-voltage relationships for BK_i and BK_s currents in WT or β2 KO SCN neurons. Steady-state current at each voltage was normalized to the maximal current level at +90 mV. Voltage protocol same as in Fig. 2. (**a-c**) The voltage-dependence of activation does not differ between BK_i and BK_s currents in WT or β2 KO SCN neurons during the day (panel **a**; P = 0.65, factorial ANOVA) or at night (panel **b**; P = 0.34, factorial ANOVA). (**c**) Rescue of inactivation by β2N does not affect the voltage dependence of activation. *n*'s: WT, day BK_i (18), BK_s (9) and night BK_i (3) BK_s (19); β2 KO, day (20) and night (20); β2 KO + β2N$^{\Delta FIW}$, day (18); and β2 KO + β2N, day (20).

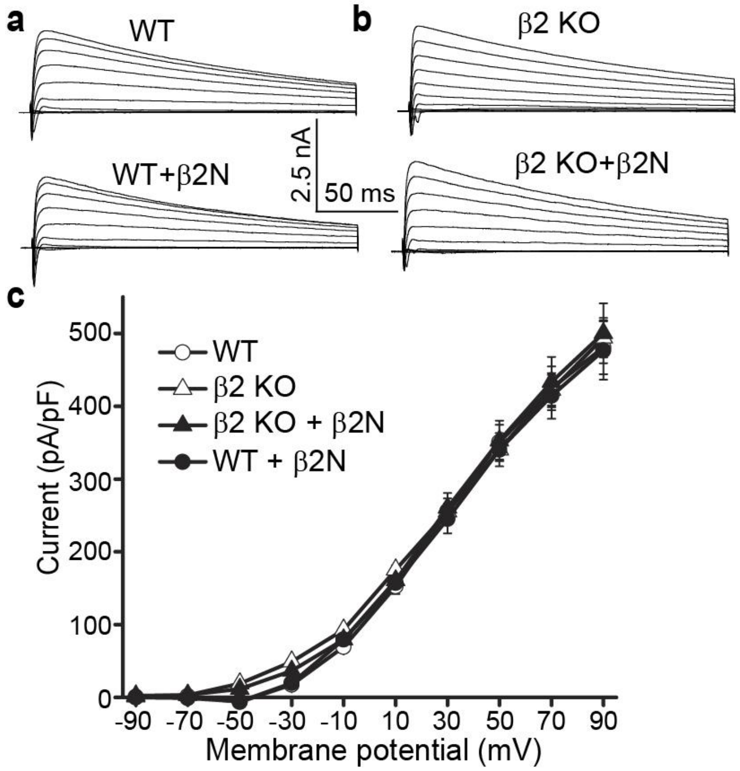

Supplementary Figure 6. β2N does not have off-target effects on non-BK currents. (**a**) Representative macroscopic current traces from WT neurons during the day (in 10 μM paxilline to block BK currents), with and without β2N. Voltage protocol same as in Fig. 2. (**b**) Representative macroscopic current traces from β2 KO neurons during the day (in 10 μM paxilline to block BK currents), with and without β2N. (**c**) Current-voltage relationships are the same with and without β2N (P = 0.88, factorial ANOVA), showing that the β2N does not cause a non-selective reduction of other voltage-activated outward K^+ currents. *n*'s: WT (27); β2 KO (20); β2 KO + β2N (20); and WT + β2N, night (20).

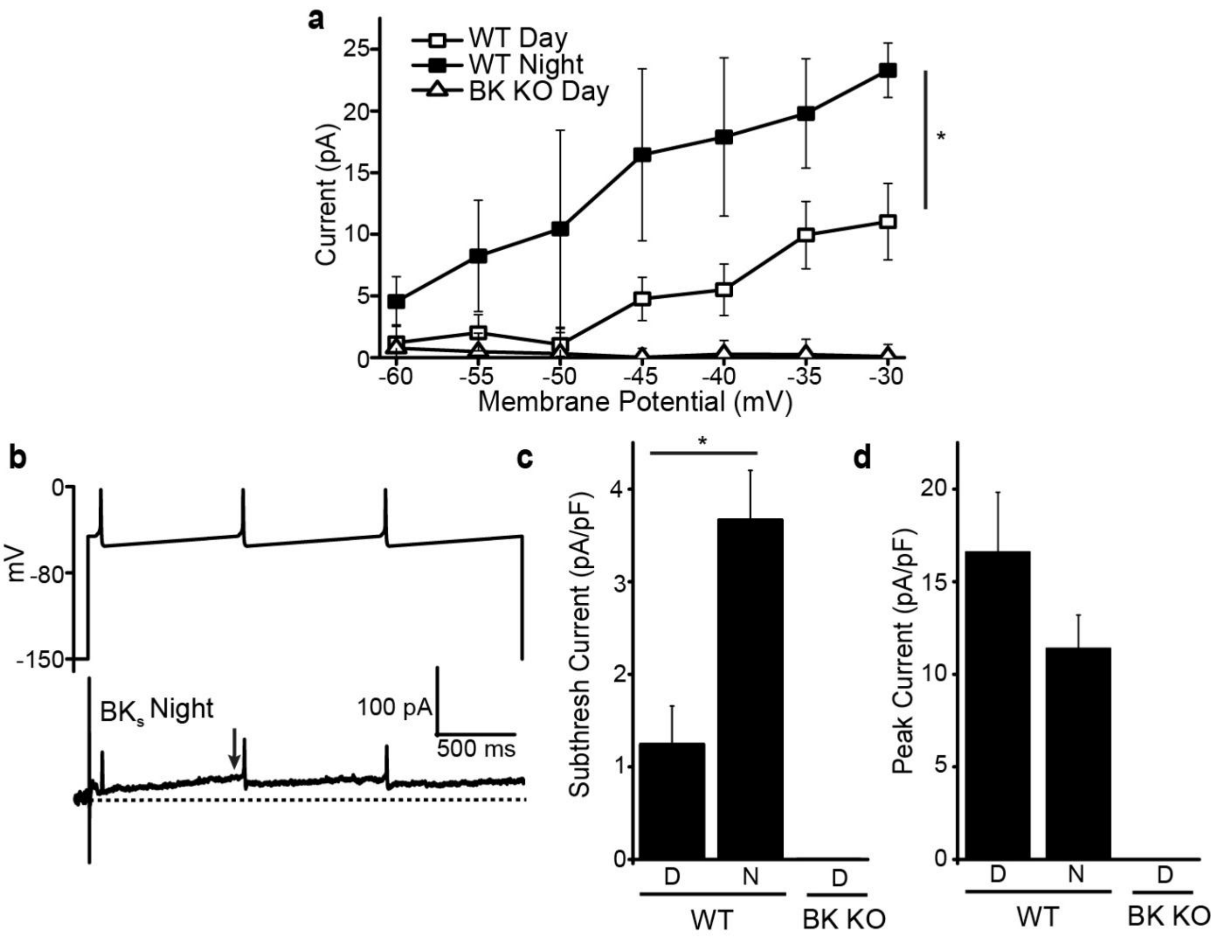

Supplementary Figure 7. Steady-state and action potential evoked BK$_s$ currents are larger at night compared to daytime BK currents in SCN neurons.

(**a**) Current-voltage relationship in response to a subthreshold voltage step protocol (–60 to –30 mV in 5 mV steps) applied to WT neurons at night (all BK$_s$), WT neurons during the day (BK$_i$ and BK$_s$ from Fig. 10b), and the BK α subunit knockout (BK KO)[1]. (**b**) Night time action potential commands (top) were used to elicit BK currents (bottom) from BK$_s$ neurons. Baseline membrane potential, –51 mV; Peak, 0.9 mV; $t_{1/2}$, 6.1 ms; and AHP/antipeak, –57 mV. Arrow, subthreshold current level in (c). (**c-d**) Average subthreshold BK current density from the interspike interval (c) or at the peak of the action potential (d). *n*'s: WT/Day (14), WT/Night (5), and BK KO (3).

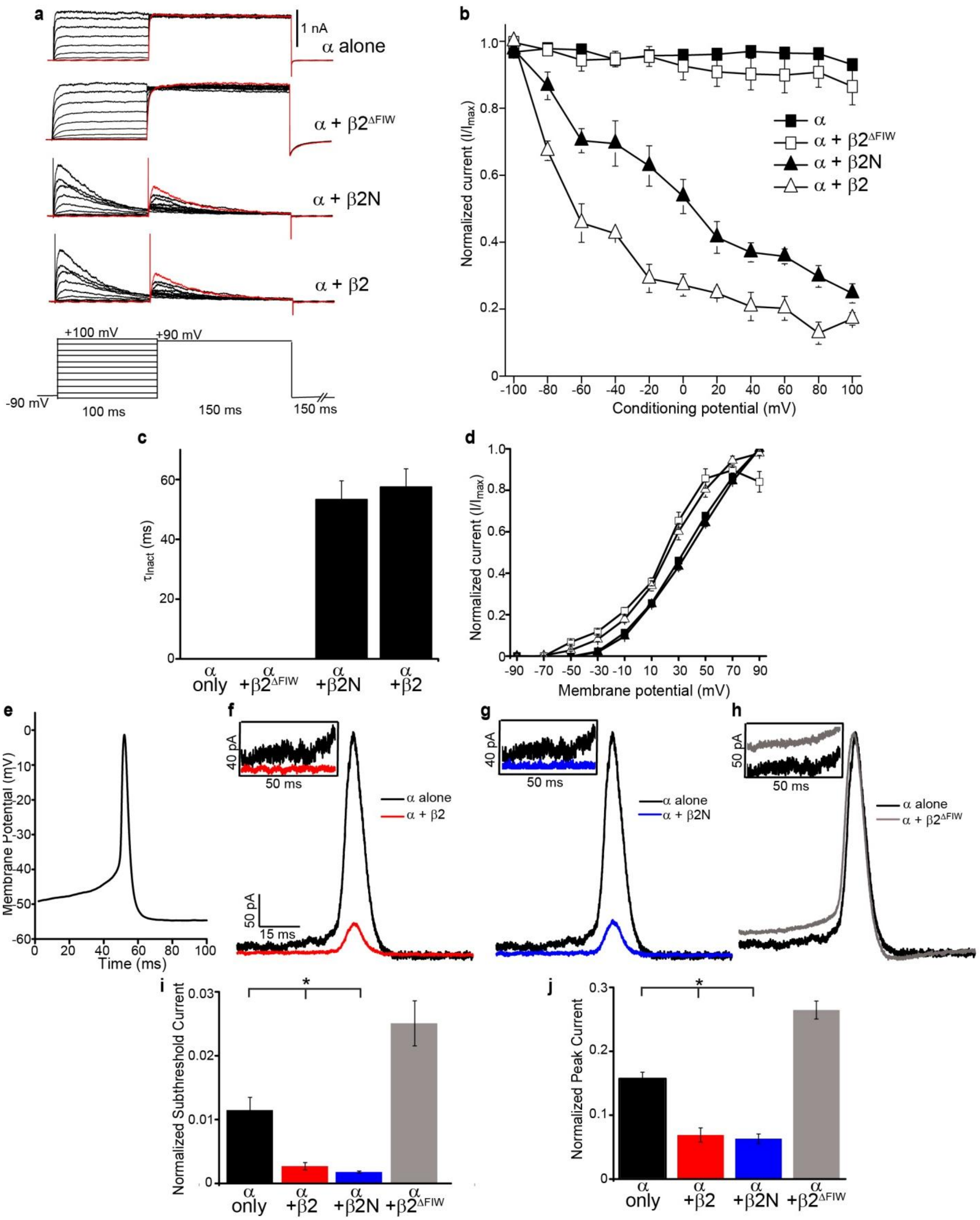

Supplementary Figure 8. The effect of inactivation on BK currents in heterologous cells. The daytime SCN variant BK_{SRKR} (ref. 2) was transfected into HEK293 cells. (**a**) Representative

macroscopic current traces from cells expressing BK_{SRKR} channels (α-only), α+β$2^{\Delta FIW}$ (co-expressed with the intact β2 subunit containing the FIW deletion), α+50 μM β2N (in the patch pipette), or α+β2 (co-expressed with the intact subunit). Patches were stepped through a conditioning protocol as depicted. (**b**) To account for differences in expression levels between cells, the peak current from the +90 mV step was normalized to the maximal current for the patch and the I/I_{max} value was plotted as a function of the conditioning potential. The BK current evoked by the maximally activating +90 mV step was reduced in conditions with inactivation (α+β2 and α+β2N) compared to conditions without (α only and α+β$2^{\Delta FIW}$). (**c-e**) A standard voltage protocol (Fig. 2a) was applied to the patches, followed by a daytime action potential waveform (from a WT BK_i neuron, as in Fig. 10c). (**c**) τ_{inact} values from the current at +90 mV. (**d**) I/I_{max} versus current relationship showing the intact β2 subunit or β$2^{\Delta FIW}$ left-shifted the voltage-dependence of activation to more hyperpolarized potentials. However, the isolated β2N peptide cannot cause this effect. (**e**) A single daytime action potential command was delivered to patches. (**f-h**) BK currents elicited by the daytime action potential command in the indicated conditions. Insets: subthreshold BK current prior to the action potential evoked current. (**i**) Average subthreshold current level normalized to the current at +90 mV. Conditions with inactivation reduce the subthreshold current, similar to native SCN neurons (Fig. 10c-d). (**j**) Peak current elicited by the action potential command normalized to the current at +90 mV. Co-expression of β$2^{\Delta FIW}$ increased AP-evoked current compared to α-only, while inactivation from the β2 subunit or β2N caused a reduction in current. *$P < 0.05$, Bonferroni post-hoc.

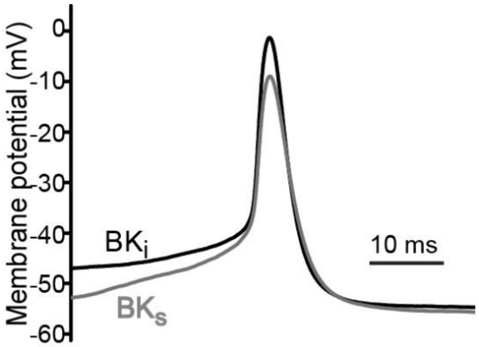

Supplementary Figure 9. Representative BK_i (black) and BK_s (gray) action potential waveforms from WT neurons during the day. Action potential parameters were determined from the average of all events in a 10 s sweep.

BK_i (*n*=17 neurons): Threshold, –41.4 ± 0.6 mV, Peak, 49.7 ± 2.5 mV, $t_{1/2}$, 5.0 ± 0.5 ms, AHP, –4.6 ± 1.3 mV

BK_s (*n*=10): Threshold, –38.2 ± 0.7 mV, Peak, 47.4 ± 3.7 mV, $t_{1/2}$, 6.1 ± 0.1 ms, AHP, –4.4 ± 0.8 mV

Supplementary Table 1: Summary of circadian behavioral rhythms

	WT	*β2 KO*	WT	*β4 KO*
**LD**	(*n* = 10)	(*n* = 10)	(n=8)	(n=8)
**τ (hrs)**	24.0 ± 0.01	24.0 ± 0.02	24.0 ± 0.01	24.0 ± 0.02
**χ^2 amp**	1730 ± 90	1410 ± 170	1865 ± 44	1930 ± 82
**DD**	(*n* = 15)	(*n* = 15)	(n=8)	(n=8)
**τ (hrs)**	23.8 ± 0.08	23.7 ± 0.05	23.9 ± 0.03	23.9 ± 0.03
**χ^2 amp**	2853 ± 146*	2099 ± 167*	1894 ± 142	1916 ± 117
**FFT rPSD (x10^{-2})**	17 ± 1*	10 ± 2*	15 ± 1	16 ± 1
**α (hrs)**	11.6 ± 0.3	11.6 ± 0.3	12.6 ± 0.2	12.5 ± 0.4
**Bout Length (mins)**	184.7 ± 17.9	164.9 ± 11.9	211.7 ± 21.1	240.4 ± 16.5
**Counts/Bout**	6553 ± 1124	6129 ± 1359	9974 ± 1164	11507 ± 999
**Bouts/Day**	3.0 ± 0.2	3.5 ± 0.2	3.0 ± 0.2	2.8 ± 0.2
**Total counts**	18371 ± 2682	18800 ± 3278	29173 ± 2320	31373 ± 1800
**α counts**	17858 ± 2628	18003 ± 3260	28489 ± 2231	30585 ± 1757
**ρ counts**	425 ± 69*	837 ± 176*	697 ± 155	777 ± 137
**Light pulse, CT16**	(*n* = 15)	(*n* = 15)		
**Phase shift (hrs)**	-1.8 ± 0.1*	-2.3 ± 0.1*		
**Phase Advance,+6L**	(*n* = 10)	(*n* = 10)		
**Days to re-entrain**	5.1 ± 0.3*	3.9 ± 0.4*		

Data are presented as mean ± s.e.m. For WT versus β2 KO, $*p < 0.05$, unpaired *t*-tests. LD, light-dark; DD, constant darkness; FFT, fast Fourier transform; rPSD, relative power spectral density; CT16, circadian time 16 h (4 hours after activity onset during constant darkness); +6L, 6 h phase advance of the light:dark cycle at lights on.

Supplementary Discussion

Circadian behavior in β2 KO mice

Circadian behavior was assessed in WT, β2 KO, and β4 KO mice by recording running wheel activity. All mice entrained to a 12:12 light:dark cycle normally and responded to light pulses, suggesting loss of β2 does not affect light input to the SCN pacemaker[3]. Mice were then placed in constant darkness to assess the intrinsic circadian period and amplitude (Supplementary Table 1). The circadian period of wheel running was not different in β2 KO compared to WT. However, like SCN action potential activity, the circadian amplitude was significantly reduced using either χ^2 periodogram analysis (amplitude of the circadian peak) or a Fast Fourier Transform (relative power of the circadian peak, 0.04-0.042 cycles/hr). The reduced circadian behavioral amplitudes were due to increased activity in the inactive (ρ) period. In contrast, the length of the active period (α), activity counts, and number of consolidated bouts were similar between β2 KO and WT. We found no differences in circadian behavior between β4 KO and WT mice.

Comparison to other transgenic alterations in BK channel activity generates an allelic series in the severity of circadian rhythm disruption of $KCNMA1^{-/-}$ > β2 KO > $Tg\text{-}BK^{R207Q}$. The behavioral phenotype was less severe in β2 KO mice compared to BK α subunit KO ($KCNMA1^{-/-}$) mice, since ~5% of the latter were completely arrhythmic[1]. β2 KOs did not have the locomotor hyperactivity or ataxia previously observed in $KCNMA1^{-/-}$ either, assessed by total wheel activity counts (Supplementary Table 1). However, compared to a gain-of-function BK channel transgenic mouse line ($Tg\text{-}BK^{R207Q}$)[4] that also exhibits remarkably similar daytime BK current levels and circuit phenotype to the β2 KO en face, the β2 KOs had a more significant circadian disruption that included a significant reduction in the circadian behavioral amplitude.

This suggests that increasing daytime BK current due to loss of the β2 'switch' mechanism is more detrimental than an equivalent increase in BK current level by other mechanisms. It is possible that β2 expression marks a more critical type of oscillator that plays a more significant role in patterning circadian behavior. It further suggests that loss of the suppressive effect of the BK channel on nighttime firing in SCN (*KCNMA1*$^{-/-}$) is more detrimental than aberrant gain of a suppressive effect due to the BK channel during the day (β2 KO and *Tg-BK*^{R207Q}).

Increased daytime BK expression reduces inactivation

If the nighttime increase in BK α subunit expression is required to overcome β2-mediated inactivation (see Main Text Discussion), then aberrantly increasing daytime BK expression would also be predicted to reduce inactivation in the SCN. To test this, we examined BK inactivation in *Tg-BK*^{R207Q} SCN neurons, which express higher levels of a gain-of-function BK channel subunit during the day[4]. *Tg-BK*^{R207Q} SCN neurons have increased BK current during the day, and we found this was due to an increase in the fractional current compared to WT (average I_{BK}/I_{BKpeak}: 0.75 ± 0.4 pF, $P < 0.05$ t-test; Supplementary Fig. 2a). This result suggests that inactivation can be 'diluted' by increasing the expression of the α subunit, consistent with increased τ_{inact} values for *Tg-BK*^{R207Q} BK currents (115 ± 40 ms) compared to WT (Supplementary Fig. 2c). This data is consistent with a model where the normal nighttime increase in BK expression is required the switch to BK_s currents, via a decrease the ratio of β:α at night.

Effect of inactivation on subthreshold and action potential-evoked BK currents in HEK293 cells

To corroborate our results in SCN neurons showing the effects of inactivation on subthreshold and action potential-evoked BK currents, we transfected BK channel constructs into HEK293 cells to examine BK currents in a heterologous system that only expresses only BK channels. In patches from HEK293 cells, subthreshold and action potential-evoked BK currents were significantly reduced in conditions with inactivation ($\alpha/\beta 2N$ or $\alpha/\beta 2$) compared to conditions without inactivation (α only or $\alpha/\beta 2^{\Delta FIW}$)(Supplementary Fig. 8e-j). These data in HEK293 cells, which express only BK channels, fully recapitulate the results obtained from SCN neurons (Fig. 10), providing complementary evidence that inactivation is the central mechanism that reduces daytime BK current.

Furthermore, other gating effects imparted by the β2 subunit can be ruled out, in terms of contributing to the reduced subthreshold and action potential evoked BK currents. Elimination of inactivation in the $\alpha/\beta 2^{\Delta FIW}$ condition, which retains the left-shifted voltage-dependence of activation (Supplementary Fig. 8d) but cannot cause inactivation, actually enhanced subthreshold and action potential evoked current compared to α only (Supplementary Fig. 8h). In contrast, this effect was opposite to the result observed in BK_i neurons, which have decreased subthreshold and action potential-evoked BK current (Fig. 10c-e). This data provides parallel evidence that inactivation, not the other aspects of β2's effects on gating, is the predominant gating property that determines β2's effect on excitability in SCN.

Supplementary References

1 Meredith, A. L. *et al.* BK calcium-activated potassium channels regulate circadian behavioral rhythms and pacemaker output. *Nature Neuro.* **9**, 1041-1049 (2006).

2 Shelley, C., Whitt, J. P., Montgomery, J. R. & Meredith, A. L. Phosphorylation of a constitutive serine inhibits BK channel variants containing the alternate exon 'SRKR'. *J Gen Physiol* **142**, 585-598 (2013).

3 Grimes, W. N., Li, W., Chavez, A. E. & Diamond, J. S. BK channels modulate pre- and postsynaptic signaling at reciprocal synapses in retina. *Nat. Neuro.* **12**, 585-592 (2009).

4 Montgomery, J. R., Whitt, J. P., Wright, B. N., Lai, M. H. & Meredith, A. L. Mis-expression of the BK K^+channel disrupts suprachiasmatic nucleus circuit rhythmicity and alters clock-controlled behavior. *Am. J. Physiol- Cell Physiol.* **304**, C299-311 (2013).
